# Effectiveness of prenatal predictors for congenital diaphragmatic hernia on survival to discharge and extracorporeal membrane oxygenation requirement: a retrospective cohort study

**DOI:** 10.1007/s00383-026-06677-z

**Published:** 2026-10-20

**Authors:** Emily Rogers, Jack Le Vance, Shashi Naidu, Anju Singh, Ingo Jester, Leo Gurney, Max Pachl

**Affiliations:** 1 Birmingham Medical School, College of Medicine and Health, University of Birmingham, Birmingham, B15 2TT UK; 2 Department of Applied Health Sciences, University of Birmingham, Birmingham, B15 2TT UK; 3 Birmingham Women’s and Children’s NHS Foundation Trust, Birmingham, B15 2TG UK; 4 Hospital Tuanku Jaafar Seremban, Jalan Rasah, 70300 Seremban, Negri Sembilan Malaysia; 5 Institute of Cancer and Genomic Sciences, College of Medicine and Health, University of Birmingham, Birmingham, B15 2TT UK

**Keywords:** Congenital diaphragmatic hernia, Prenatal, Prognostic, Ultrasound, Survival, ECMO

## Abstract

**Introduction:**

Congenital diaphragmatic hernia (CDH) is associated with high mortality and morbidity. Antenatal counselling enables parents to prepare for the postnatal outcomes and relies on effective prognostic tools. Ultrasound (USS) predictors including observed/expected lung head ratio (o/e LHR) and liver position, are used independently, or combined into severity classifications. The aim was to evaluate the association between USS predictors, and severity classifications with survival to discharge and extracorporeal membrane oxygenation (ECMO) requirement in prenatally diagnosed CDH at Birmingham Women’s and Children’s Hospital (BWC).

**Methods:**

CDH patients from BWC with dates of birth between 1st December 2013–1st December 2023 were retrospectively collected. Study inclusion required o/e LHR measurement between 22- and 32-weeks gestational age (GA.) Comparisons between groups was assessed by Fisher exact test and Mann-Whitney U test. Logistic regression was performed to determine prognostic ability, with area under curve (AUC) calculated.

**Results:**

91 eligible patients were identified, which included 82 left-sided, and 9 right-sided CDH. Overall, 55% survived to discharge and 13% required ECMO. In the left CDH cohort, 57% of infants survived to discharge, and 15% required ECMO. O/e LHR, liver position and the combined predictive model including o/e LHR and liver position were predictive of survival to discharge with good prognostic ability (AUC 0.769, AUC 0.703, and AUC 0.823, respectively). O/e LHR, liver position, and the combined predictive model were not predictive of ECMO requirement. In the right CDH patients, 33% survived to discharge, O/e LHR and liver position were not predictive of survival to discharge.

**Conclusion:**

O/e LHR, liver position and the combination of these predictors, predicted survival to discharge in prenatally diagnosed left CDH with the combined model, the most predictive particularly in the isolated left CDH sub-group. Further analysis into effective predictors for ECMO requirement, and for right and complex left CDH is required.

**Supplementary Information:**

The online version contains supplementary material available at https://doi.org/10.1007/s00383-026-06677-z.

## Introduction

Congenital diaphragmatic hernia (CDH) is a posterolateral defect of the diaphragm, allowing passage of abdominal viscera into the thorax, leading to the development of pulmonary hypoplasia. Following birth, this can exhibit as signs of respiratory insufficiency and persistent pulmonary hypertension (PPHN), requiring intensive care [1]. CDH can be classified according to several characteristics: location of the defect, 84% occurring in the left hemithorax [2], isolated or complex and any coexisting congenital anomalies [3, 4].

CDH is a rare serious congenital abnormality, with a global prevalence of 2–3 in 10,000 livebirths. CDH, when accompanied with other congenital anomalies has poorer prognosis with perinatal mortality up to 50% [5]. Given this, prenatal detection with risk stratification and subsequent postnatal treatment remains paramount to ensure beneficial perinatal outcomes [5–7].

Prenatal detection has significantly improved due to prenatal screening ultrasound (USS), with 2/3 of CDH cases detected by the second trimester [8–10]. Prenatal predictors measured on USS and magnetic resonance imaging (MRI), along with genetic testing and echocardiography guide clinician and parental decisions around pregnancy continuation, termination of pregnancy (TOP), prenatal interventions, and postnatal outcomes [11]. USS predictors such as lung-head ratio (LHR), observed/expected (o/e) LHR, and liver position are the standards in prenatal prediction [12]. Despite evidence supporting use of MRI predictors, these techniques are limited by accessibility of relevant MRI sequencing [12].

A classification of CDH, incorporates o/e LHR and liver position to prognosticate survival to discharge (StD) [12]. Left CDH can be classified as mild, moderate and severe, illustrated in Table S1 [12–14].

Right-sided CDH can be classified into severe and non-severe, with severe classified as o/e LHR ≤ 50% and associated with a survival rate of just 20% [15].

In this study, we retrospectively investigated prenatal and postnatal outcomes of CDH cases at our tertiary centre. We stratified by severity classification, incorporating o/e LHR and liver position, assessing any differences between prenatal and postnatal outcomes. Further analysis studying isolated and complex stratified by side of defect was undertaken to compare differences in prenatal and postnatal outcomes and efficacy of prenatal predictors for prognostication.

## Methods

### Study design

This retrospective cohort study was conducted at Birmingham Women’s and Children’s NHS Foundation Trust (BWC), a regional CDH centre with a tertiary fetal medicine centre and neonatal intensive care unit at one site and a surgical unit with paediatric intensive care at another. Inclusion criteria included all neonates born 1 st December 2013 to 31 st December 2023 with a prenatal diagnosis of CDH receiving perinatal care at BWC; LHR or o/e LHR measurements and liver position acquired between 22- and 32- weeks of gestation as recommended [12]. Cases were included if stored images of adequate quality were available for retrospective calculation of prenatal predictors. Participants were excluded due to insufficient reporting of postnatal data for outcome assessment.

### Image assessment

Antenatal ultrasounds were performed using Voluson E8 or E10 Expert (GE Healthcare Austria GmbH & Co. OG, Zipf, Austria) with a 2–6 MHz transabdominal probe by maternal-fetal medicine specialists. LHR was measured at the four-chamber view of the heart as standard [16]. O/e LHR was obtained from scan reports, or if LHR was reported, this was converted to o/e LHR using the formulae in Table S2. Calculation was dependent on the measurement technique [13].

Where CDH cases had no calculated o/e LHR or LHR, USS images were retrospectively measured if images were taken at 22–32-weeks gestation, with a transverse thorax view clearly demonstrating the heart’s four-chambers. Longest diameter measurements were performed by an independent fetal medicine specialist (SH) and ratified by a second specialist (LG). Acquired measurements were subsequently converted to o/e LHR using the equations in Table S2. The average o/e LHR and liver position were stratified into three subgroups for left CDH (mild, moderate and severe), using criteria in Table S1. Right CDH was classified into severe, defined as o/e LHR ≤ 50%, or non-severe.

### Data collection and outcomes

Maternal and fetal electronic records were used to retrospectively collect clinical outcome data. Liver position was defined as ‘liver-up,’ defined as any volume of liver situated within the thorax or ‘liver-down’.

The primary outcomes were neonatal StD from BWC and requirement of extracorporeal membrane oxygenation (ECMO).

Criteria for initiation of ECMO at BWC included:

- Period of adequate oxygenation with ventilation achieved with optimal management.
- Current inability to maintain pre-ductal saturation > 85% of post-ductal saturations > 70% despite optimal ventilation and management of pulmonary hypertension.
- Systemic hypotension, resistant to fluid and inotropic therapy with a urine output < 0.5 ml/kg/hr over 12–24 h.
- Episodes of pulmonary hypertensive crisis.
- Pulmonary hypertensive crisis resulting in a cardiac arrest.
- Impaired right or left ventricular function.
- Inability to sustainably wean adrenaline < 0.1 mcg/kg/minute or noradrenaline < 0.1mcg/kg/minute.
- Failure to wean inotropes, HFOV or iNO at 10 days.

Maternal demographic variables of maternal age (years), maternal body mass index (BMI), parity, smoking history, prenatal corticosteroid usage, mode of delivery (vaginal, emergency caesarean section (EMCS), elective caesarean section (ELCS)) were also collected.

Neonatal and fetal outcomes included gestational age (GA) at diagnosis, birthweight, GA at birth, presence of congenital abnormalities, hydrops fetalis, polyhydramnios, fetal growth restriction (FGR), small for gestational age (SGA). Other characteristics included Apgar score at 5 min, total neonatal intensive care unit (NICU) stay, age at surgical repair, type of surgical access (laparoscopic, open, laparoscopic converted to open), surgical repair (patch or primary closure), pulmonary hypertension (pre-operative or post-operative), total NICU stay, and total hospital stay.

Criteria for surgical readiness is for repair for CDH at BWC includes:

- Resolution of hypoxia (preductal saturation 85–95% on FiO2 ≤ 50%.)
- Blood pressure normalisation.
- Adequate tissue oxygen delivery.
- Adequate tissue perfusion.
- Stabilisation of pulmonary hypertension, weaned from iNO.

Participants were stratified based on defect laterality and isolated CDH, classified as the absence of any additional structural or chromosomal anomalies or complex CDH, classified as one or more other major congenital anomalies including congenital heart disease, urogenital, craniofacial, and musculoskeletal defects, or recognised genetic/chromosomal conditions identified antenatally or postnatally.

### Statistical analysis

Demographic and outcome categorical data was presented numerically with percentages and continuous data presented as medians with interquartile range (IQR). Demographic and outcome data differences between left and right-sided CDH, isolated and complex, survival/no StD, and ECMO requirement (yes/no) was assessed using the Fisher exact test for categorical variables and Mann-Whitney U test for continuous variables. Isolated and complex left CDH were compared to identify any differences and outcomes were assessed between three groups, such as comparing left CDH severity classification using the X^2^ test for independence or Kruskal-Wallis Test. Dichotomous outcome data were assessed with 2 × 2 tables to calculate odds ratio (OR) and 95% confidence intervals (CI). A p value < 0.05 was considered statistically significant.

Univariable logistic regression and receiver operating characteristics (ROC) was used to investigate the deterministic power of o/e LHR and liver position at predicting StD and ECMO requirement. when cohort size permitted, using area under the curve (AUC), sensitivity, specificity, positive predictive value (PPV) and negative predictive value (NPV.) An optimal cutoff for the o/e LHR was determined empirically using Youden’s index, to maximise the sum of sensitivity and specificity [17]. Multivariable logistic regression was used on appropriate sample sizes to investigate prognostic accuracy when average o/e LHR and liver position were combined. Multivariable logistic regression was used when appropriate to investigate the predictive value of USS predictors and other significant variables, including Apgar score at 5 min, GA at diagnosis, birthweight and presence of congenital anomalies on StD. Statistical analyses were performed using IBM SPSS statistics ver. 29.0 (IBM Co., Armonk, NY, USA).

## Results

### Population characteristics

**Fig. 1:**
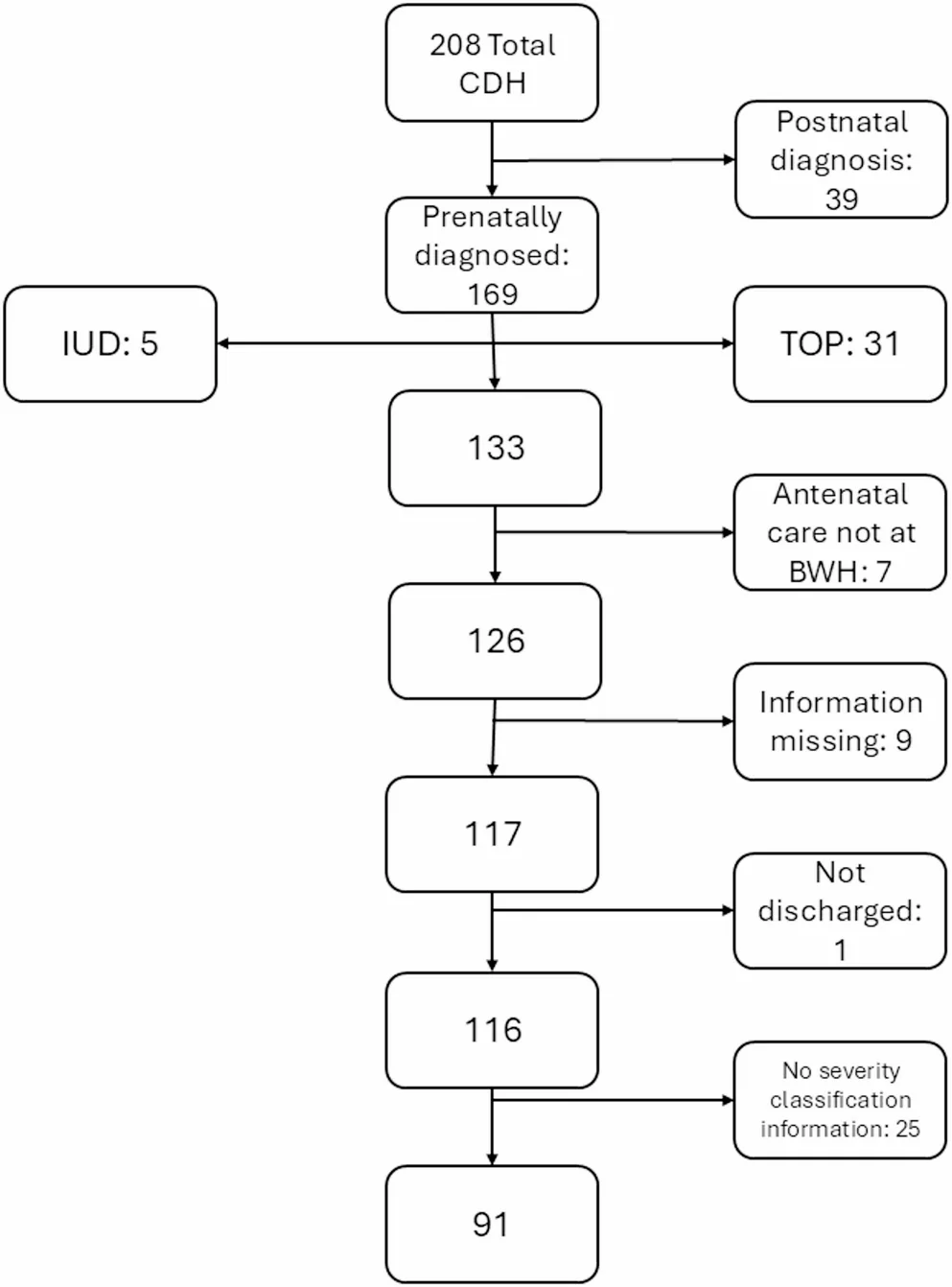
CONSORT diagram showing patient inclusion and exclusion. *IUD* Intrauterine death, *TOP* Termination of pregnancy, *BWH* Birmingham women’s hospital

208 CDH cases were retrospectively identified, with 169 cases prenatally diagnosed, (81%). Following removal of TOP (*n* = 31), intrauterine demises (*n* = 5), cases not receiving antenatal care at our trust (*n* = 7), cases with insufficient information (*n* = 9) and those without a severity classification (*n* = 25), 91 cases were included for analysis (Fig. 1).

**Fig. 2:**
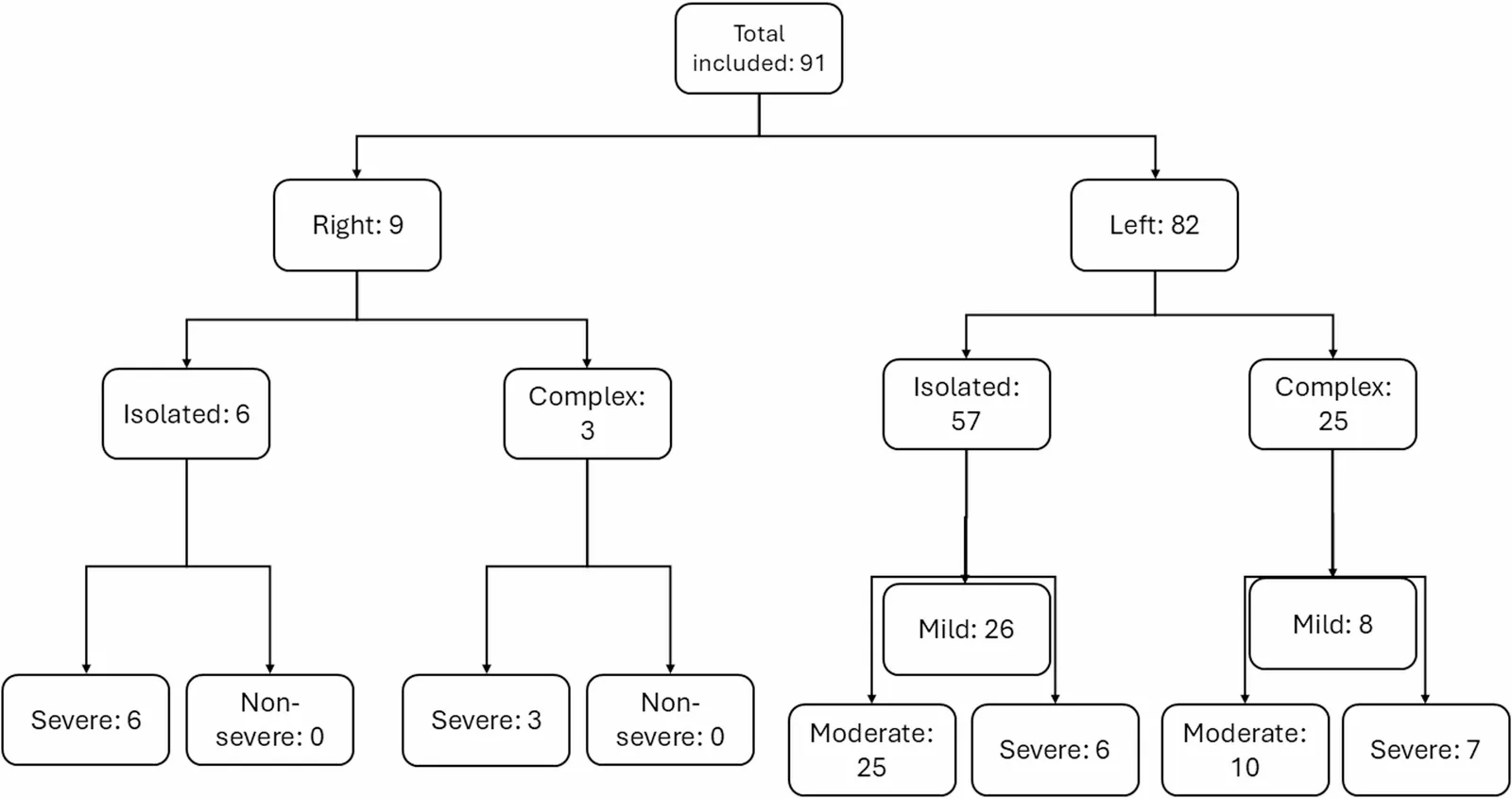
Cohort population breakdown. Breakdown into left and right, isolated, and complex and severity classifications

Eighty-two (90%) were left CDH and 9 (10%) right CDH with 31% complex (Fig. 2). 70% of left CDH and 67% right were isolated.

The complex CDH cases were classified into- craniofacial, congenital heart defects, urogenital, syndromic, musculoskeletal, and neural, with the cohort breakdown in Table S3. Cardiac comorbidities, defined as structural abnormalities identified on echocardiography, excluding patent foramen ovale, patent ductus arteriosus and atrial septal defects were identified in 10 cases (11%).

Baseline characteristics of the overall cohort including separation of left and right CDH cases are presented in Table 1. A significantly higher GA at birth (*p* = 0.025), higher average o/e LHR (*p* = 0.027) and lower proportion of intrathoracic liver (*p* = 0.025) was found in the left CDH cohort.

**Table 1:** Baseline characteristics of overall cohort, left and right CDH

				Overall		Left CDH		Right CDH		P value
				n		n		n		
Male sex (n (%))				91	43 (47)	82	40 (49)	9	3 (33)	0.716
Ethnicity (n (%))			White	91	50 (55)	82	46 (56)	9	4 (44)	0.726
			Asian		26 (29)		22(27)		4 (44)	0.270
			Mixed		6 (7)		6 (7)		0 (0)	1.000
			Black		2 (2)		2 (2)		0 (0)	1.000
			Other		7 (8)		6 (7)		1 (11)	0.530
GA at birth (median (IQR))				91	38^+ 1^ (37^+ 2^ − 39^+ 3^)	82	38^+ 2^ (37^+ 2^ − 39^+ 3^)	9	37^+ 2^ (35–38^+ 1^)	0.025
Birthweight (grams) (median (IQR))				91	2900 (2608–3345)	82	2900 (2690–3360)	9	2645 (1970–3100)	0.120
GA at diagnosis (median (IQR))				91	21^+ 5^ (20^+ 5^ - 24^+ 2^)	82	21^+ 4^ (20^+ 5^ − 24^+ 2^)	9	22^+ 3^ (21^+ 5^ – 23^+ 3^)	0.108
Average o/e LHR (median (IQR))				91	39 (28–49.5)	82	39 (29–51)	9	24 (22–39)	0.027
Intrathoracic liver n (%)				90	59 (66)	81	50 (62)	9	9 (100)	0.025
Congenital abnormalities (n (%))				91	28 (31)	82	25 (30)	9	3 (33)	1.000
Hydrops fetalis (n (%))				91	2 (2)	82	1 (1)	9	1 (11)	0.189
Polyhydramnios (n (%))				90	31 (34)	81	26 (32)	9	4 (44)	0.489
FGR (n (%))				91	10 (11)	82	8 (10)	9	2 (22)	0.257
SGA (n (%))				91	15 (16)	82	12 (15)	9	2 (22)	0.639
Maternal age (years; median (IQR))				91	29 (26–34)	82	29 (26–33)	9	33 (28–35)	0.137
Maternal BMI (median (IQR))				70	28.0 (24.4–32.0)	71	27.7 (23.9–32.3)	9	29.7 (24.6–31.5)	0.681
Nulliparous (n (%))				91	37 (41)	82	33 (40)	9	4 (44)	1.000
Maternal smoking (n (%))				89	10 (11)	80	10 (13)	9	0 (0)	0.590
Prenatal corticosteroids (n (%))				86	17 (20)	77	14 (18)	9	3 (33)	0.372
Mode of delivery (n (%))	Vaginal			91	56 (62)	82	50 (61)	9	6 (67)	1.000
	EMCS				18 (20)		16 (20)		2 (22)	1.000
	ELCS				17 (19)		16 (20)		1 (11)	1.000
Apgar score at 5 min (median (IQR))				84	8 (6–9)	76	8 (6–9)	8	7.5 (5–9)	0.437
NICU stay total (days median (IQR))				89	16 (6–26)	81	15 (7–27)	9	1 (1–19)	0.241
Surgical repair (n (%))				91	59 (65)	82	56 (68)	9	3 (33)	0.062
Neonatal age at surgical repair (days- median, (IQR))				59	9 (5.5–13)	56	9 (5.75–13.25)	3	8 (6–10.5)	0.732
Type of surgical access (n (%))		Laparoscopic		59	15 (25)	56	15 (27)	3	0 (0)	0.559
		Open			34 (58)		31 (55)		3 (100)	0.265
		Laparoscopic converted to open			8 (14)		8 (14)		0 (0)	1.000
		No data			2 (3)		2 (4)		0 (0)	0.074
Type of surgical repair (n (%))		Patch		59	44 (75)	56	44 (79)	3	3 (100)	1.000
		Primary closure			10 (17)		10 (18)		0 (0)	1.000
		No data			2 (3)		2 (4)		0 (0)	1.000
PPHN (n (%))				88	74 (84)	80	67 (84)	8	7 (88)	0.620
Pre-op PPHN (n (%))				88	72 (82)	80	66 (83)	8	6 (75)	0.633
Post-op PPHN (n (%))				55	17 (31)	51	15 (29)	4	2 (50)	0.573
Total NICU days post-surgery (median (IQR))				50	20 (13–28)	47	21 (13–28.5)	3	19 (18.5–33)	0.482
Total hospital days (median (IQR))				47	42 (26.5–59)	44	39.5 (25.75–59)	3	43 (40.5–69)	0.492

*CDH* congenital diaphragmatic hernia, *ELCS* elective caesarean section, *EMCS* emergency caesarean section, *FGR* fetal growth restriction, *GA* gestational age, *IQR* interquartile range, *o/e LHR* observed/expected lung-head ratio, *n *number, *NICU* neonatal intensive care unit, *PPHN* persistent pulmonary hypertension, *post-op* postoperative, *pre-op* preoperative, *SGA* small for gestational age

### Outcomes

Overall, 55% of neonates StD. When stratified by CDH type, 57% and 33% of left-and right-sided CDH survived respectively. ECMO was required in 13% of the cohort, 15% of left-sided and none in the right-sided cohort (Table 2).

**Table 2:** Outcomes of overall cohort and left and right CDH subgroups

		Overall		Left		Right		P value
		n		n		n		
StD (n (%))		91	50 (55)	82	47 (57)	9	3 (33)	0.290
Death	Pre-op death (n (%))	41	31 (76)	35	26 (74)	6	5 (83)	0.264
	Post-op death (n (%))		10 (24)		9 (26)		1 (17)	0.528
	Age at death (days, median, (IQR))		2 (1–13)		3 (1–15.5)		1 (1–1)	0.079
ECMO requirement (n (%))		89	12 (13)	81	12 (15)	9	0 (0)	0.591
Length of time on ECMO (days; median (IQR))		89	8 (6–14)	81	8 (6–14)	/	/	/

*StD Survival to discharge, ECMO* extracorporeal membrane oxygenation, *IQR* interquartile range, *n* number

### Left CDH

#### Survival to discharge

Table 3 compares characteristics of left CDH cases which StD versus those that did not. Higher birthweight, later gestational age at diagnosis, absence of congenital abnormalities, higher Apgar score at 5 min were positively associated with StD (*p* = 0.018, *p* = 0.030, *p* < 0.001 and *p* < 0.001 respectively). All left-sided CDH neonates who StD underwent surgical repair (*p* < 0.001), with open patch repair being the only surgical technique used in those who do not StD (*p* < 0.001). PPHN was poorly associated with StD (*p* < 0.001), with 100% babies who did not survive having PPHN. 

**Table 3:** Comparison of characteristics between cohort which StD and those who did not survive to discharge in left CDH cohort

			StD		No StD		P value
			n		n		
Male sex (n (%))			47	26 (55)	35	14 (40)	0.188
Ethnicity (n (%))		White	47	28 (60)	35	18 (51)	0.893
		Asian		11 (23)		11 (31)	
		Mixed		4 (9)		2 (6)	
		Black		1 (2)		1 (3)	
		Other		3 (6)		3 (9)	
GA at birth (median (IQR))			47	38^+ 2^ (37^+ 5^ − 39^+ 4^)	35	38^+ 1^ (36^+ 3^ − 39^+ 3^)	0.729
Birth weight grams (median (IQR))			47	3170 (2765–3410)	35	2830 (2445–3183)	0.018
GA at diagnosis (median. IQR)			47	22^+ 1^ (20^+ 6^ – 26^+ 2^)	35	21^+ 2^ (20^+ 3^ – 22^+ 3^)	0.030
Average o/e LHR (median (IQR))			47	43 (37.5–54.5)	35	28 (23.5–37.5)	< 0.001
Intrathoracic liver (n (%))			47	21 (45)	34	29 (85)	< 0.001
Severity classification (n (%))	Mild		47	28 (60)	35	6 (17)	< 0.001
	Moderate			17 (36)		18 (51)	
	Severe			2 (4)		11 (31)	
Congenital abnormalities (n (%))			47	6 (13)	35	19 (54)	< 0.001
Hydrops fetalis (n (%))			47	0 (0)	35	1 (3)	0.427
Polyhydramnios (n (%))			47	13 (28)	34	14 (41)	0.238
FGR (n (%))			47	4(9)	35	4 (11)	0.718
SGA (n (%))			47	7 (15)	35	6 (17)	1.000
Maternal age (years; median (IQR))			47	30 (26–34)	35	28 (25–33)	0.350
Maternal BMI (median (IQR))			37	27.8 (23.8–32.0)	34	27.7 (24.7 - 33.8)	0.881
Nulliparous (n (%))			47	17 (36)	35	16 (46)	0.495
Maternal smoking (n (%))			45	6 (13)	35	4 (11)	1.000
Prenatal corticosteroids (n (%))			46	10 (22)	31	4 (13)	0.381
Mode of delivery (n (%))	Vaginal		47	25 (53)	35	25 (71)	0.191
	Emergency Caesarean section			10 (21)		6 (17)	
	Elective Caesarean section			12 (26)		4 (11)	
Apgar score at 5 min (median (IQR))			45	9 (7–9)	31	7 (4–8)	< 0.001
Surgical repair (n (%))			47	47 (100)	35	9 (26)	< 0.001
Neonatal age at surgical repair (days; median (IQR))			47	9 (5.5–11)	9	14 (8–17)	0.165
Type of surgical access (n (%))	Laparoscopic		47	15 (32)	9	0 (0)	0.018
	Open			22 (47)		9 (100)	
	Laparoscopic converted to open			8 (17)		0 (0)	
	No data			2 (4)		0 (0)	
Type of surgical repair (n (%))	Patch		47	36 (77)	9	8 (89)	< 0.001
	Primary closure			10 (21)		0 (0)	
	No data			1 (2)		1 (11)	
PPHN (n (%))			47 47 47	34 (72)	33 33 7	33 (100)	< 0.001 < 0.001 0.001
Pre-op PPHN (n (%))				33 (70)		33 (100)	
Post-op PPHN (n (%))				9 (19)		6 (86)	

 Cases who StD were associated with a higher average o/e LHR and intrabdominal liver position (*p* < 0.001 and *p* < 0.001 respectively).

**Fig. 3:**
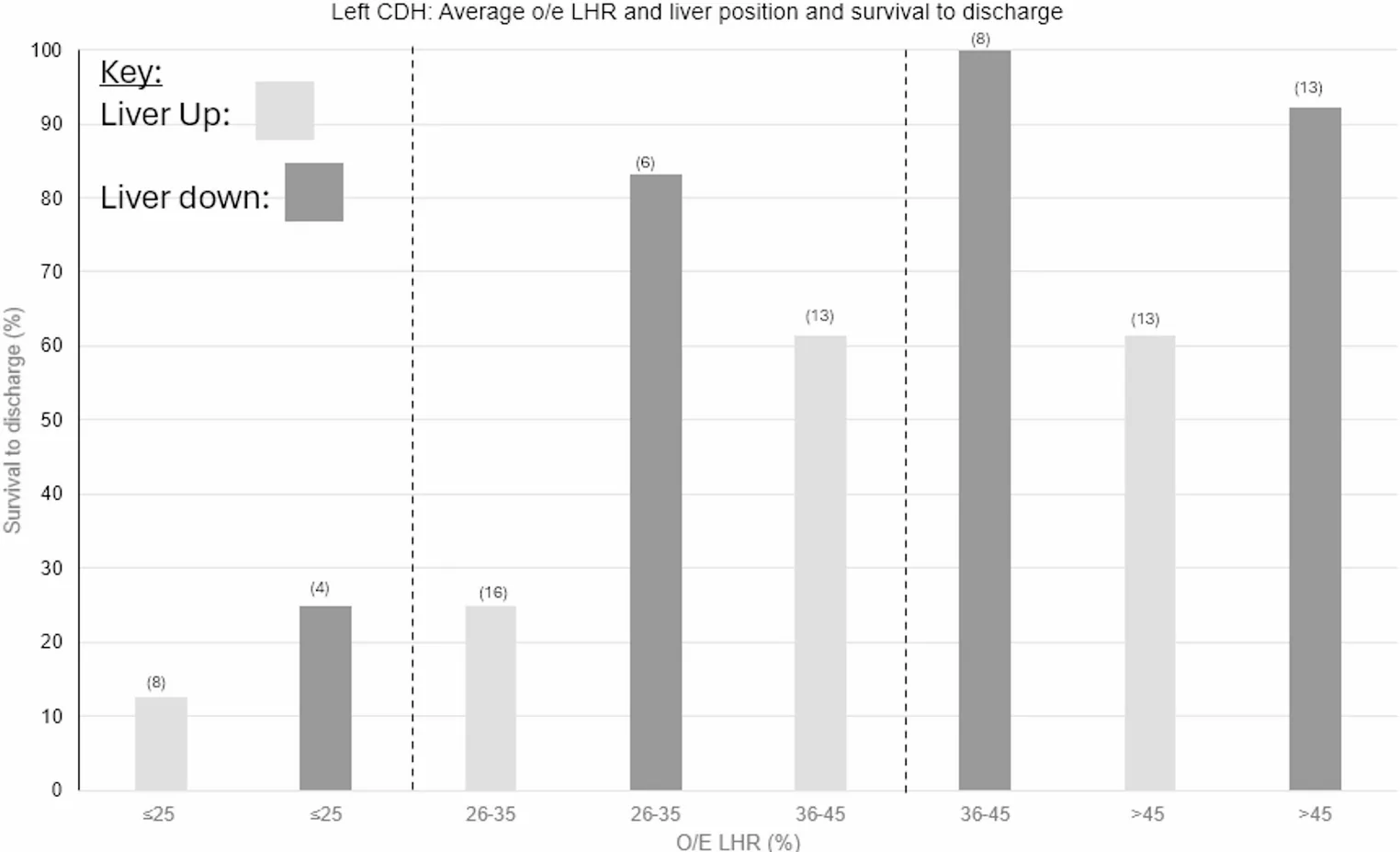
StD (%) for o/e LHR category with liver up and liver down separated, for left CDH cohort (n) represents total number of patients within each sub-group. Dotted lines demonstrate severity classification cut-offs *CDH* congenital diaphragmatic hernia, *o/e LHR* observed/expected lung-head ratio

Binomial logistic regression found o/e LHR correctly identified 73.2% of infants who StD (78.7% sensitivity, 65.7% specificity, 75.5% PPV, 70% NPV, AUC 0.769 (95% CI: 0.655–0.882)). An optimal cut-off for o/e LHR was 39.5% (sensitivity 70.2%, specificity 80%), suggesting those with a o/e LHR above 39.5% have a significant likelihood of StD. Intrabdominal liver position correctly identified 67.9% of infants who StD (55.3% sensitivity, 85.3% specificity, 83.9% PPV and 58% NPV, AUC 0.703 (95% CI: 0.589–0.817)). The association between o/e LHR and liver position with StD is demonstrated in Fig. 3. Birthweight, GA at diagnosis, Apgar score at 5 min and isolated vs. complex were statistically significant on univariate logistic regression (*p* = 0.045, *p* = 0.018, *p* < 0.001 and *p* < 0.001.

Of the independent variables which were significantly significant on univariate logistic regression, several analyses were undertaken to identify if an optimal model was most predictive for StD. Initially, prenatal information (isolated vs. complex, average o/e LHR, liver position, GA at diagnosis) was modelled, (Table 4). The AUC for this model was 0.886 (0.808–0.964) suggesting excellent discrimination.

**Table 4:** Multivariate regression analysis for prenatal predictors influences on StD in left CDH

		Survival	
	n (%) or median (IQR)	OR (95% CI)	P value
Average o/e LHR	39 (29–51)	1.051 (1.010–1.095)	0.015
Intrathoracic liver	50 (62)	9.513 (2.164–41.808)	0.003
Complex CDH		0.093 (0.023–0.382)	< 0.001
Gestational age at diagnosis	21^+ 4^ (20^+ 5^ − 24^+ 2^)	1.106 (0.924–1.323)	0.271

*CI* confidence interval, *IQR* interquartile range, *o/e LHR* observed/expected lung-head ratio, *n* number, *OR* odds ratio

Of the variables significantly associated with StD individually, multivariable logistic regression analysis demonstrated that the independent significant predictors were average o/e LHR, liver position, and Apgar score at 5 min (Table 5). Birthweight and gestational age at diagnosis were not significant.

**Table 5:** Multivariate regression analysis for StD in left CDH

		Survival	
	n (%) or median (IQR)	OR (95% CI)	P value
Average o/e LHR	39 (29–51)	1.055 (1.019–1.092)	0.002
Intrathoracic liver	50 (62)	7.181 (2.367–21.783)	< 0.001
Birthweight (g)	2900 (2690–3360)	1.001 (1.000–1.002)	0.045
Gestational age at diagnosis	21^+ 4^ (20^+ 5^ − 24^+ 2^)	1.175 (1.028–1.344)	0.018
Apgar score at 5 min	8 (6–9)	1.764 (1.306–2.381)	< 0.001

*CI* confidence interval, *IQR* interquartile range, *o/e LHR* observed/expected lung-head ratio, *n* number, *OR* odds ratio

#### Severity classification

Multivariable logistic regression combining o/e LHR and liver position was statistically significant (ꭓ^2^(2) = 24.023.) This model suggested severity classifications correctly identified 70.4% of infants who StD with 70.2% sensitivity, 70.6% specificity, 76.7% PPV, 63.2% NPV and AUC 0.823 (95% CI: 0.729–0.916).

Lower birthweight was significantly associated with more severe CDH classifications (*p* = 0.022), a higher incidence of fetal growth restriction (FGR) and small for gestational age (SGA) found in the severe CDH group (*p* = 0.005 and p-0.041, respectively). Additionally, higher Apgar scores were more frequently associated with mild CDH cases (*p* = 0.044).

The proportion of patients undergoing surgical repair was significantly associated with severity classification (*p* = 0.006), with patients from the mild CDH group more likely to have surgery. The type of surgical access and surgical repair were associated with severity classification (*p* = 0.035 and *p* = 0.022, respectively), with a higher proportion of open surgery (80%) and primary closure (40%) in the severe CDH group.

PPHN was associated with severity classification, with prevalence overall and pre-operatively being significantly different between groups (*p* = 0.003 and *p* = 0.009, respectively), with higher prevalence of PPHN reported in the severe left CDH group.

#### ECMO requirement

Later gestational age at birth was significantly associated with ECMO requirement (*p* = 0.036). Average o/e LHR and liver position were not significantly associated with ECMO requirement (*p* = 0.138 and *p* = 0.351) in left CDH.

#### Subgroup analysis

As the absence of congenital abnormalities were significantly associated with StD, subgroup analysis of isolated and complex CDH was undertaken.

Table 6 demonstrates the outcomes of the isolated and complex left CDH subgroups.

**Table 6:** Outcomes of isolated and complex left CDH cohort

		Isolated CDH		Complex CDH		P value
		n		n		
StD (n (%))		57	41 (72)	25	6 (24)	< 0.001
Death	Pre-op death (n (%))	16	11 (69)	19	15 (79)	< 0.001
	Post-op death (n (%))		5 (31)		4 (21)	0.172
	Age at death (days, median, (IQR))		8.5 (1.75–19.75)		2 (1–8)	0.953
ECMO requirement (n (%))		57	9 (16)	24	3 (13)	0.034
Length of days on ECMO (median (IQR))		9	8 (6–9)	3	13 (9.5–15.5)	0.270

*CDH* congenital diaphragmatic hernia, StD survival to discharge, *ECMO* extracorporeal membrane oxygenation, *IQR* interquartile range, n number, *post-op *postoperative, *pre-op* preoperative

### Isolated left CDH

#### Survival to discharge

Higher Apgar score at 5 min, surgical repair, earlier timing of surgical repair and lower incidence of PPHN were significantly higher in infants who StD (*p* = 0.009, *p* < 0.001, *p* = 0.034 and *p* = 0.013 respectively).

In the isolated left CDH cohort, o/e LHR was significantly associated with StD (*p* = 0.02). Univariable logistic regression was statistically significant ꭓ^2^(1) = 7.408 and correctly identified 78.9% of infants who StD with 31.3% sensitivity, 97.6% specificity, 78.4% PPV and 83.3% NPV, AUC 0.760 (95% CI: 0.596–0.924). The optimal cutoff to predict likelihood for StD for o/e LHR was 37.5% (73.2% sensitivity and 82.2% specificity).

**Fig. 4:**
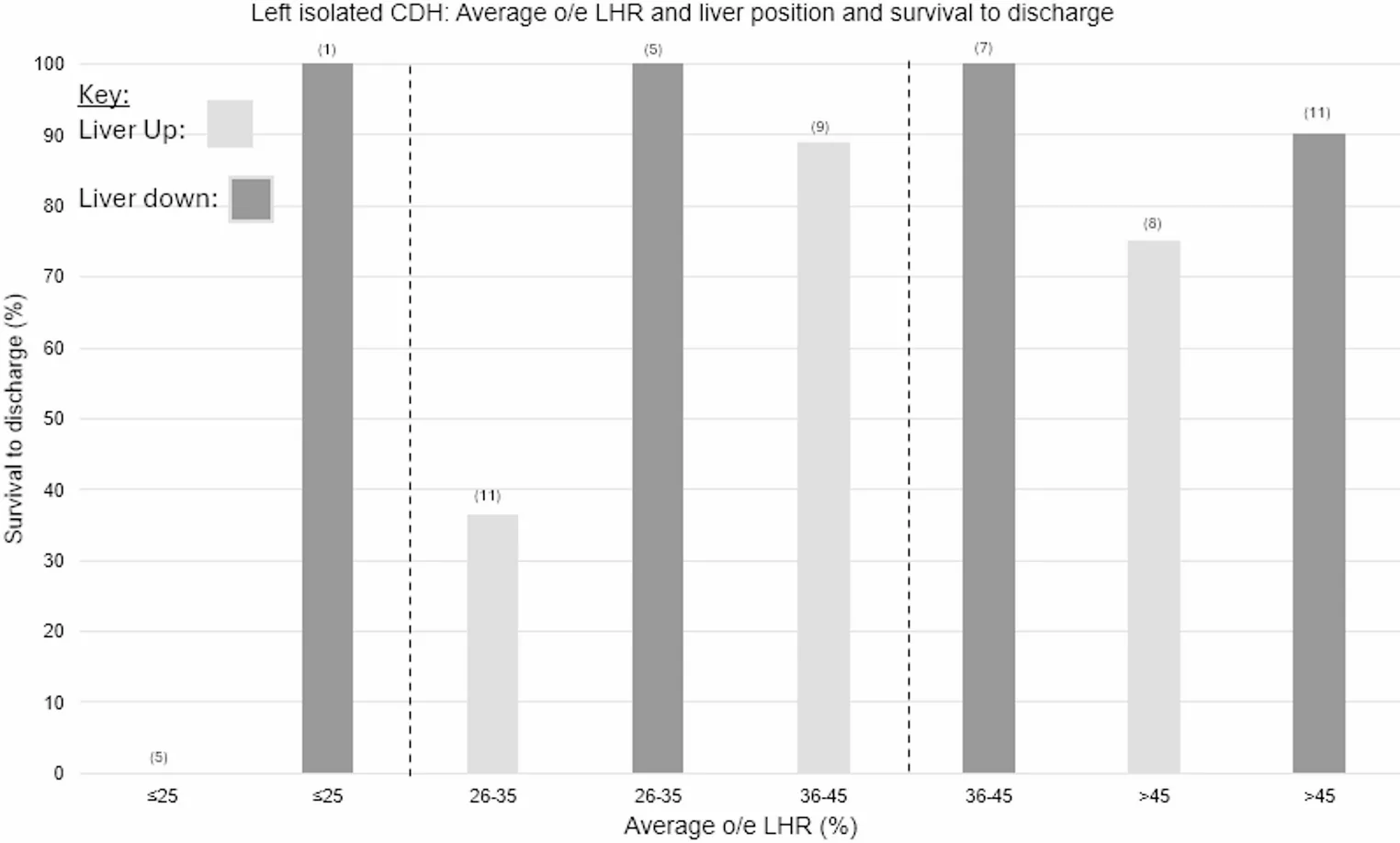
Survival to discharge (%) for o/e LHR category with liver up and liver down separated, for left isolated CDH cohort. (n) represents total number of patients within each sub-group. Dotted lines demonstrate severity classification cut-offs. *CDH* congenital diaphragmatic hernia; *o/e LHR* observed/expected lung-head ratio

Liver position was significantly associated with StD (*p* < 0.001). Univariable logistic regression was statistically significant ꭓ^2^(1) = 13.884 and correctly identified 71.9% of infants who StD, with 0% sensitivity, 100% specificity, 71.9% PPV and 0% NPV and AUC 0.749 (95% CI: 0.620–0.878). Figure 4 depicts the association between average o/e LHR and liver position and StD.

#### Severity classification

Multivariable logistic regression was statistically significant, ꭓ^2^(2) = 18.044, and correctly identified 86% of cases, with 90.2% sensitivity, 75% specificity, 90.2% PPV, 75% NPV and AUC 0.873 (95% CI: 0.747–0.998). On multivariate logistic regression, liver position was the only independent predictor of StD (*p* = 0.012).

88% mild, 68% moderate and 17% severe isolated left CDH StD. Type of surgical repair was the only statistically significant difference between severity classifications (*p* = 0.028). Average o/e LHR and liver position combined into severity classification was significantly associated with StD (*p* = 0.002).

#### ECMO requirement

ECMO was required in 8%, 16% and 50% of mild, moderate and severe cases of isolated left CDH respectively.

Higher gestational age at birth was significantly associated with requirement for ECMO (*p* = 0.042). No significant difference between average o/e LHR or liver position and ECMO requirement (*p* = 0.073 and *p* = 1.000, respectively). Severity classification was significantly associated with ECMO requirement (*p* = 0.038). However, no multivariable logistic regression could be performed due to the limited cohort size.

### Complex left CDH

#### Survival to discharge

Apgar score at 5 min and the proportion of neonates who underwent surgical repair were statistically significant between those who StD and those who did not (*p* = 0.020 and *p* = 0.001 respectively). The presence of post-operative PPHN was significantly associated with StD (*p* = 0.048), with all those having surgery and subsequently not surviving, having experienced PPHN. Average o/e LHR was significantly associated with StD (*p* = 0.043), but liver position was not significant (*p* = 0.307).

The proportion of patients undergoing surgical repair was the only significant difference between severity classification subgroups (*p* = 0.047). StD rates were significantly different between severity classification groups (*p* = 0.007), with 63% of mild, 0% moderate and 14% severe cases surviving to discharge.

#### ECMO requirement

Prenatal predictors were not associated with ECMO requirement individually (o/e LHR *p* = 1.000 and intrathoracic liver position *p* = 0.155) or when combined (*p* = 0.927).

### Right CDH

33% right CDH StD, and no cases required ECMO (Table 1). Table S4 compares those who StD, showing no association between average o/e LHR, liver position and StD. However limited conclusions could be drawn due to the small sample size (*n* = 9).

## Discussion

Overall, 55% of neonates in this study StD, and 13% required ECMO. In left CDH cohort, 57% StD, with a significant association between higher o/e LHR and intrabdominal liver position and StD, particular in the isolated left CDH cohort. In complex left CDH cohort, a higher o/e LHR was significantly associated with StD. In right CDH cohort, o/e LHR and intrabdominal liver position were not significantly associated with StD. The prenatal predictors were not associated with ECMO requirement.

### Prenatal diagnosis

The prenatal prediction (81%) was higher than historical data (50–60%), suggesting BWC are effective at identifying CDH, which could explain the high cohort mortality rate since prenatal diagnosis is associated with worse outcomes [10, 18, 19].

### Survival rate

The StD rate (55%), was lower than other multicentre studies, reporting survival rates of 60%−75% [5, 20]. The mortality rate of this cohort is not representative of total mortality at BWC.

The survival rate of left CDH (82% mild, 49% moderate, 15% severe) was lower than 2022 CDH data [21]. 2023 CDH guidelines report a ≤ 30% survival for severe left CDH should be used in antenatal counselling [12]. Therefore, the survival in the left cohort was comparable to historic data and current guidelines but lower than latest data. The smaller sample size may give minor discrepancies in our results, continued prospective analysis will improve the validity of these results.

The survival rate of the right CDH cohort was lower than that reported in small retrospective studies, however due to the small right CDH cohort size limited conclusions can be drawn [22–24]. 

The reduced survival rates in the study could be explained by the inclusion of complex CDH cases, frequently disregarded from survival analysis. The mortality rate represents overall mortality over the entire study period, failing to reflect changes in clinical practice including increased availability of ECMO and other temporal improvements, a trend demonstrated in other CDH studies [20].

Another explanation for reduced StD rates could be reduced ECMO utility with studies reporting a 34% ECMO requirement, compared to the 13% utility in this population [23, 25–27]. This could be explained by inclusion of complex CDH within the cohort, reducing the candidates for ECMO, however there was reduced ECMO use in the isolated cohort suggesting this is not the predominant explanation [21, 28, 29]. Differences in clinical practice between centres could explain differences in utility of ECMO [25, 29, 30]. BWC follow trust specific ECMO guidelines, however variability remains since clinical judgement is the cornerstone of ECMO use [31]. Furthermore, ECMO was not onsite at the beginning of the study period, potentially resulting in reduced use or increased patient exclusion. ECMO can improve outcomes in CDH, particularly those with severe pulmonary hypoplasia, although evidence supporting its use is controversial as comparable survival rates are demonstrated when utilising minimal ECMO [29, 32, 33].

### Prenatal predictors

This study demonstrated that o/e LHR and liver position were independent predictors for StD in left CDH cases [23, 25, 26, 34–37]. The discrimination (AUC 0.769) and cut-off (39.5%) of o/e LHR for StD for left CDH was comparable or higher than findings of similar studies [21, 26, 34]. The predictive value of liver position for StD (AUC 0.703) was comparable to previous findings [34].

Prenatal predictors were not associated with ECMO requirement, dissimilar to previous findings [21]. Further statistical analysis was limited by the small cohort receiving ECMO, reducing clinical conclusions regarding the use of severity classifications for ECMO prediction.

Subgroup analysis found prenatal predictors were useful in isolated left CDH but had limited value in complex left CDH. Limited research into prenatal predictors for complex CDH means it is unknown if this finding is consistent with other tertiary centres. Prenatal predictors had no value in predicting ECMO requirement for isolated or complex left CDH, dissimilar to previous studies [25, 26].

Prenatal predictors were not associated with StD in right CDH, comparable to large study’s findings but dissimilar to findings in smaller retrospective studies [22, 23, 38]. This demonstrates the need for further research into this subgroup of patients with worse outcomes though it is challenging to study due to the small sample size [24].

### Clinical implications

This study adds to the evidence supporting o/e LHR and liver position as predictive tools for StD in left CDH, particularly isolated left CDH. This study highlighted the limited evidence for prognostic tools for right sided CDH, identifying a future research area. It demonstrates significant survival differences between isolated and complex CDH, and that prenatal predictors cannot be adopted in complex CDH, giving clinicians an antenatal challenge when attempting to prognosticate these patients.

### Strengths and limitations

The study involved a rigorous process of identifying suitable participants, reducing the likelihood of missed cases. Extensive data collection allowed for wide-ranging analysis providing region specific outcome data to aid prenatal and postnatal counselling. Separated analysis of isolated and complex CDH cases provided insight into outcome differences and the applicability of prenatal predictors for each group.

This study is limited by the retrospective study design, and limited cohort size. The sub-group size made it harder to reach conclusions about right and complex CDH. The limited right CDH cohort size limited appropriate statistical tests and the ability to draw appropriate conclusions. Variability in management and interventions between tertiary centres limit generalisability of findings to other centres. The definition of liver position might overstate the occurrence of intrathoracic liver as some scans reported only a small portion of liver within the thorax; however, this study reported them as positive. This might have resulted in more patients within higher severity classifications, limiting comparisons between cohorts which define liver position differently [14]. ECMO was recorded as a binary outcome so the timing of when ECMO was given was not recorded. Therefore, this outcome measure is limited as it is unclear whether ECMO was utilised as surgical support ECMO or following surgical intervention. Multivariate analysis was unable to be performed due to the small sample size requiring ECMO. Therefore, the conclusions made about this outcome are significantly limited and have reduced generalisability to wider populations.

### Future considerations

This study suggests further investigation of prenatal predictors would be valuable. A long-term prospective study investigating stomach position and MRI findings as predictors of outcomes could determine superior predictors, whilst improving the power and efficacy of current results [21, 23, 25, 26, 34, 39–41]. Continued research could guide changes in prediction protocol and allow better prognostication of right and complex left CDH and prediction of ECMO requirement.

## Conclusion

This research demonstrated that o/e LHR and liver position are effective prenatal tools to determine survival to discharge in left CDH, however there is additive value in combining these into severity classification. Therefore, severity classifications should be used when antenatally counselling isolated left CDH patients. The prenatal predictors had limited value predicting ECMO requirement, however limited statistical analysis of this cohort was performed due to the small sample size. Due to the small cohort of right CDH no conclusions can be drawn for the utility of prenatal prediction for survival to discharge in this cohort. The StD rate in prenatally diagnosed CDH was lower than previous estimates, so cautious estimates should be used when counselling parents on likelihood of survival to ensure appropriate prediction.

## Supplementary Information

Below is the link to the electronic supplementary material.

Supplementary Material 1

## Data Availability

No datasets were generated or analysed during the current study.

## Abbreviations

CDH: Congenital diaphragmatic hernia
USS: Ultrasound
TOP: Termination of pregnancy
StD: Survival to discharge
LHR: Lung-head ratio
o/e LHR: Observed-to-expected lung-head ratio
BWC: Birmingham women’s hospital
ECMO: Extracorporeal membrane oxygenation
BMI: Body mass index
EMCS: Emergency caesarean section
ELCS: Elective caesarean section
GA: Gestational age
FGR: Fetal growth restriction
SGA: Small for gestational age
NICU: Neonatal intensive care unit
OR: Odds ratio
CI: Confidence interval
ROC: Receiver operating curve
AUC: Area under the curve
PPV: Positive predictive value
NPV: Negative predictive value
