# Supplementary material for: Effectiveness of prenatal predictors for congenital diaphragmatic hernia on survival to discharge and extracorporeal membrane oxygenation requirement: a retrospective cohort study
Source: Pediatr Surg Int. 2026 Oct 20;42(1):447. doi: 10.1007/s00383-026-06677-z (PMC13683475; doi:10.1007/s00383-026-06677-z)
Supplement: Supplementary file 1 [file 383_2026_6677_MOESM1_ESM.docx]

**Supplementary Information**

**Table S1:** USS Criteria for severity classification of left CDH and survival rate (%)

| **Severity** | **USS Criteria** | **Survival Rate (%)** |
| --- | --- | --- |
| Mild | O/E LHR 36%-45% and ‘liver down’ OR  O/E LHR >45%, irrespective of liver position. | ≥85 |
| Moderate | O/E LHR 26-35%, any liver position OR  O/E LHR 36-45% and ‘liver up’ | 40-60 |
| Severe | O/E LHR ≤25%, irrespective of liver position | ≤30 |

O/E LHR, observed/expected lung-head ratio.

**Table S2:** Formulae for expected fetal lung area-to-head circumference ratio (LHR)

| **Lung area measurement** | **Right LHR in left CDH** | **Left LHR in right CDH** |
| --- | --- | --- |
| Longest diameter method | -3.4802 + (0.3995 x GA) – (0.0048 x GA²) | -2.5957 + (0.3043 x GA) – (0.0042 x GA²) |
| Anteroposterior diameter method | -3.1597 + (0.3615 x GA) – (0.0041 x GA²) | -1.0224 + (0.1314 x GA) – (0.0011 x GA²) |
| Tracing method | -2.3271 + (0.27 x GA) – (0.0032 x GA²) | -1.4994 + (0.1778 x GA) – (0.0021 x GA²) |

CDH, congenital diaphragmatic hernia; GA, gestational age; LHR, lung-head ratio. Formulae for left- and right-sided CDH using different methods of measurement of the lung area, based on gestational age (GA) in weeks with decimals (18).

**Table S3:** Congenital abnormalities present in complex CDH cohort, note some babies were identified as having several abnormalities

| Congenital abnormality | Complex CDH. |
| --- | --- |
| Craniofacial | 6 |
| CHD | 10 |
| Urogenital | 10 |
| Chromosomal | 9 |
| Musculoskeletal | 3 |
| Neural | 5 |

**Table S4:** Comparison of characteristics of right CDH between those who StD and those who did not survive to discharge.

|  | | | **StD** | | **No StD** | | **P value** |
| --- | --- | --- | --- | --- | --- | --- | --- |
|  |  |  | n |  | n |  |  |
| Male sex (n (%)) | | | 3 | 1 (33) | 5 | 2 (40) | 1.000 |
| Ethnicity (n (%)) | | White | 3 | 0 (0) | 6 | 4 (67) | 0.105 |
|  |  | Asian |  | 2 (67) |  | 2 (33) |  |
|  |  | Mixed |  | 0 (0) |  | 0 (0) |  |
|  |  | Black |  | 0 (0) |  | 0 (0) |  |
|  |  | Other |  | 1 (33) |  | 0 (0) |  |
| GA at birth (median (IQR)) | | | 3 | 38^+1^ (34^+5^ - 38^+4^) | 6 | 36^+4^ (35^+1^ - 37^+4^) | 0.714 |
| Birth weight grams (median (IQR)) | | | 3 | 3100 (2535 - 3315) | 6 | 2323 (1565 - 2836) | 0.381 |
| GA at diagnosis (median. IQR) | | | 3 | 21^+5^ (21^+4^ – 26^+3^) | 6 | 22^+5^ (22^+2^ – 23^+2^) | 0.905 |
| Average o/e LHR (median (IQR)) | | | 3 | 22 (20.5 - 34.5) | 6 | 26.5 (22.5 - 26.5) | 0.905 |
| Intrathoracic liver (n (%)) | | | 3 | 3 (100) | 6 | 6 (100) | N/A |
| Severity classification (n (%)) | Severe | | 3 | 3 (100) | 6 | 6 (100) | N/A |
|  | Non-severe | |  | 0 (0) |  | 0 (0) |  |
| Hydrops fetalis (n (%)) | | | 3 | 0 (0) | 6 | 1 (17) | 1.000 |
| Polyhydramnios (n (%)) | | | 3 | 3 (100) | 6 | 1 (17) | **0.048** |
| FGR (n (%)) | | | 3 | 0 (0) | 6 | 2 (33) | 0.500 |
| SGA (n (%)) | | | 3 | 0 (0) | 6 | 2 (33) | 0.500 |
| Maternal age (years; median (IQR)) | | | 3 | 36 (32 - 37.5) | 6 | 32.5 (28.25 - 34.5) | 0.262 |
| Maternal BMI (median (IQR)) | | | 3 | 28.2 (24.8 - 29.4) | 6 | 30.6 (25.9 - 31.7) | 0.381 |
| Nulliparous (n (%)) | | | 3 | 1 (33) | 6 | 3 (50) | 1.000 |
| Maternal smoking (n (%)) | | | 3 | 0 (0) | 6 | 0 (0) | N/A |
| Prenatal corticosteroids (n (%)) | | | 3 | 1 (33) | 6 | 2 (33) | 1.000 |
| Mode of delivery (n (%)) | Vaginal | | 3 | 2 (67) | 6 | 4 (67) | 0.687 |
|  | Emergency Caesarean section | |  | 1 (33) |  | 1 (17) |  |
|  | Elective Caesarean section | |  | 0 (0) |  | 1 (17) |  |
| Apgar score at 5 minutes (median (IQR)) | | | 2 | 9 (9 - 9) | 6 | 7 (3 - 8) | 0.143 |
| Surgical repair (n (%)) | | | 3 | 3 (100) | 6 | 0 (0) | 0.012 |
| Neonatal age at surgical repair (days; median (IQR)) | | | 3 | 8 (6-10.5) | 0 | 0 (0) | N/A |
| Type of surgical access (n (%)) | Laparoscopic | | 3 | 0 (0) | 0 | 0 (0) | N/A |
|  | Open | |  | 3 (100) |  | 0 (0) |  |
|  | Laparoscopic converted to open | |  | 0 (0) |  | 0 (0) |  |
|  | No data | |  | 0 (0) |  | 0 (0) |  |
| Type of surgical repair (n (%)) | Patch | | 3 | 3 (100) | 0 | 0 (0) | N/A |
|  | Primary closure | |  | 0 (0) |  | 0 (0) |  |
|  | No data | |  | 0 (0) |  | 0 (0) |  |
| PPHN (n (%)) | | | 3  3  3 | 2 (67) | 5  5  0 | 5 (100) | N/A  N/A  N/A |
| Pre-op PPHN (n (%)) | | |  | 1 (33) |  | 5 (100) |  |
| Post-op PPHN (n (%)) | | |  | 1 (33) |  | 0 (0) |  |
